# Dose-Dependent Immunomodulation of Human Dendritic Cells by the Probiotic *Lactobacillus rhamnosus* Lcr35

**DOI:** 10.1371/journal.pone.0018735

**Published:** 2011-04-18

**Authors:** Bertrand Evrard, Sophie Coudeyras, Annie Dosgilbert, Nicolas Charbonnel, Josette Alamé, Arlette Tridon, Christiane Forestier

**Affiliations:** 1 CHU Clermont-Ferrand, Laboratoire d'Immunologie, Clermont-Ferrand, France; 2 Clermont Université, Université d'Auvergne, UFR Médecine-Pharmacie, EA4233, Laboratoire d'Immunologie, Clermont-Ferrand, France; 3 Clermont Université, Université d'Auvergne, UFR Pharmacie, Laboratoire de Bactériologie, Clermont-Ferrand, France; Charité, Campus Benjamin Franklin, Germany

## Abstract

The response of the immune system to probiotics remains controversial. Some strains modulate the cytokine production of dendritic cells (DCs) *in vitro* and induce a regulatory response, while others induce conversely a pro-inflammatory response. These strain-dependent effects are thought to be linked to specific interactions between bacteria and pattern recognition receptors. We investigated the effects of a well characterized probiotic strain, *Lactobacillus rhamnosus* Lcr35, on human monocyte-derived immature DCs, using a wide range of bacterial concentrations (multiplicity of infection, MOI, from 0.01 to 100). DNA microarray and qRT-PCR analysis showed that the probiotic induced a large-scale change in gene expression (nearly 1,700 modulated genes, with 3-fold changes), but only with high doses (MOI, 100). The upregulated genes were mainly involved in immune response and identified a molecular signature of inflammation according to the model of Torri. Flow cytometry analysis also revealed a dose-dependent maturation of the DC membrane phenotype, until DCs reached a semi-mature state, with an upregulation of the membrane expression of CD86, CD83, HLA-DR and TLR4, associated with a down-regulation of DC-SIGN, MR and CD14. Measurement of the DC-secreted cytokines showed that Lcr35 induced a strong dose-dependent increase of the pro-Th1/Th17 cytokine levels (TNFα, IL-1β, IL-12p70, IL-12p40 and IL-23), but only a low increase in IL-10 concentration. The probiotic *L. rhamnosus* Lcr35 therefore induce a dose-dependent immunomodulation of human DCs leading, at high doses, to the semi-maturation of the cells and to a strong pro-inflammatory effect. These results contribute to a fuller understanding of the mechanism of action of this probiotic, and thus of its potential clinical indications in the treatment of either infectious or IgE-dependent allergic diseases.

## Introduction

Dendritic cells (DCs) are antigen presenting cells that play a critical role in the orchestration of the adaptative immune response by inducing both tolerance and immunity [1], [2]. The current paradigm is that this dual role is the result of the division of the total DC population into a network of DC subsets with distinct functions [3], [4]. Immature DCs reside in peripheral tissues, such as the gut mucosa, where they sense the microenvironment via pattern recognition receptors (PRRs), including toll-like receptors (TLRs) and C-type lectin receptors (CLRs), which recognize pathogen products called pathogen-associated molecular patterns (PAMPs) [5]. This antigenic stimulation triggers a DC maturation process with up-regulation or down-regulation of membrane molecules CD83, CD86, HLA-DR and DC-SIGN respectively, and cytokine production. The activation of the DCs by several PAMPs with antagonistic or synergistic effects, through distinct PRRs, modulates their differentiation, which secondarily determines the polarization of the effector T cell responses, i.e. Th1, Th2, Th17 or T regulatory (Treg) responses [6]. An important factor in this process is cytokine production by DCs. When they produce IL-12, polarization is driven towards Th1 cells, whereas the synthesis of IL-1β, IL-6, TGFβ and IL-23 leads to Th17 cells, and that of IL-10, towards Treg cells [7], [8]. Recently, Torri *et al.* proposed a model of DCs gene expression profiling (a pattern of 54 tested genes) that can identify of the anti- or pro-inflammatory effects of tested bacteria or molecules on DC activity [9].

The human gastrointestinal tract is continuously exposed to several hundreds of commensal bacteria species from the intestinal flora. Yet, under physiological conditions, little or no inflammation occurs in the mucosal barrier. The understanding of the direct interactions between commensal bacteria and DCs is especially important to know how the immune system of the gut is locally able to distinguish them from pathogens and to elicit a tolerogenic response. Among the commensal intestinal bacteria, some strains of lactobacilli, named probiotics, are known to confer benefit to human health. The mechanisms of probiotic actions include immune modulation of DCs, but their effects on DCs maturation are not well understood and vary widely according to the bacteria used [10]. *In vitro*, some strains modulate the production of cytokines by dendritic cells (or monocytes) and induce a regulatory response by eliciting the production of IL-10, whereas others induce Th1-mediated pro-inflammatory responses through the production of IL-12 [11]–[14]. These strain-dependent effects are probably linked to interactions between specific bacterial surface structures and the PRRs, such as the DC-SIGN, a member of the CLR family, which interacts with certain strains, but not with others [13]. Nevertheless, most studies reporting immune effect of probiotics have used very different bacteria/DC ratios [11]–[14]. We cannot rule out the possibility that these differences in ratio influenced the DC maturation process and therefore their response to the bacteria. It is noteworthy that the *in vivo* effects of probiotics are generally observed after regular daily medication, suggesting a significant dose effect.

To adress this question, we investigated the effects of a well characterized probiotic strain, *Lactobacillus rhamnosus* Lcr35 [15]–[17], on human monocyte-derived immature DCs. A variety of techniques, from global gene transcription profile to expression of membrane and soluble proteins analysis, was used to characterize the DCs responses after contact with Lcr35. All assays were performed with a wide range of Lcr35 concentrations. We were thus able to observe differences according to the dose of probiotic bacteria used, and in particular a strong pro-inflammatory, pro-Th1 and pro-Th17 effects at high doses.

## Methods

### Bacterial strain and culture conditions

*Lactobacillus rhamnosus* 35 (Lcr35) was grown without agitation in De Man, Rogosa, Sharpe (MRS) medium (BD Difco™, BD diagnostics, Le Pont-De-Claix, France) at 37°C overnight. Bacterial cells were harvested by centrifugation (11,000× g for 10 min), and the pellet resuspended in DC culture medium (RPMI 1640). Optical measures (Mac Farland) were performed to adjust the final concentration of the bacterial suspension, and the exact number of colony forming units (CFU) was determined by plating serial dilutions of the inocula onto MRS plates. Before being added to the DC samples, the bacterial cells were inactivated by exposure to UV for 40 min. Successful inactivation of bacteria was assessed by plating the final suspension on agar plates.

### Ethic statement

Human cells used in this study were generated from the buffy-coat of 14 healthy volunteers obtained from the local French blood agency (Etablissement Français du Sang (EFS), Saint-Etienne). Blood donation requires the systematic information of the volunteers (article R.1221-5 of the Public Health Code, 01/12/2009 and 06/11/2006 decrees) and written informed consents were obtained by EFS from all donors involved in our study.

### *In vitro* differentiation of monocyte-derived dendritic cells

DCs were generated from peripheral blood mononuclear cells (PBMC). Briefly, PBMCs were isolated from the buffy-coat of healthy volunteers by Ficoll-Histopaque (Sigma, Saint-Quentin Fallavier, France) density gradient centrifugation. For phenotypic assays, the PBMCs were washed twice with RPMI 1640 (Cambrex Bio Science, Verviers, Belgium), and the monocytes were then isolated by adherence (2 hours). The surface of the plates (75 cm^2^ flasks, BD Falcon, Le Pont de Claix, France) was precoated with 1 µg/mL Poly-L Lysine (PLL, Sigma) for 2 h at 4°C. On the other hand for transcriptional assays, the PBMCs were resuspended in PBS supplemented with 2% FCS and 1 mM EDTA at a final concentration of 5.10^7^ cells/ml and the monocytes were purified by negative selection using the EasySep® Human Monocyte Enrichment Kit as recommended by the manufacturer (StemCell Technologies, Grenoble, France). In both cases, the monocytes were then cultured for 5 days in RPMI 1640 supplemented with 1% L-glutamine (Sigma), 10% FCS (Biowest-Abcys, Paris, France) and 0.5% penicillin-streptomycin (Sigma) and containing 500 U/mL IL4 (R&D systems, Lille, France) and 800 U/mL granulocyte-macrophage colony-stimulating factor (GM-CSF from R&D systems). After 3 days of incubation, one-half volume of fresh medium containing 2× doses of IL4 and GM-CSF was added to each well. The purity of the dendritic cells was assessed by flow cytometry analysis using a marker highly specific of the DC lineage, the DC-SIGN, and was always above 90% (figure S1).

### Transcriptional assays

On day 6, immature dendritic cells were washed twice with PBS, resuspended in fresh growth medium without antibiotic and seeded at a concentration of 10^6^ cells/cm^2^ in 24-well Nunc tissue plates. Freshly grown bacteria were washed and resuspended in PBS to obtain *L. rhamnosus* Lcr35 suspensions with concentrations of 10^9^ and 10^6^ CFU/ml. Ten microliters of bacterial suspensions, i.e 10^7^ or 10^4^ CFU, or PBS without bacteria (control) were added to each well, permitting to obtain a multiplicity of infection (MOI) of 10, 0.01 or 0 (control) respectively, and the tissue culture plates were incubated for 3 hours to allow interaction of *L. rhamnosus* Lcr35 with the cells. The DCs were rinced twice with PBS before proceeding to total RNA extraction using TRIzol® reagent as recommended by the supplier (Invitrogen) with slight modifications. Briefly, cells were homogenized and lysed by addition of 400 µl of TRIzol® reagent to each well, repetitive pipetting followed by incubation for 10 min at room temperature. Lysates were vortexed 15 s after addition of 80 µl of chloroform and then centrifuged for 15 min at 12,000× *g* at 4°C. The upper aqueous phase was washed once with chloroform. Total RNA was precipitated by addition of one volume of isopropanol, incubated for 10 min at −20°C and centrifuged for 15 min at 12,000× *g* and 4°C. The RNA pellet was washed twice with 80% ethanol, dried, and complete dissolution was obtained by addition of water, repetitive pipetting, and incubation for 15 min at 65°C. RNA samples were stored at −80°C after quantification and quality assessment using a Qubit® fluorometer with the Quant-iT™ RNA Assay Kit (Invitrogen) and a NanoDrop™ spectrophotometer (NanoDrop products, Wilmington, DE, USA).

### Microarray gene expression profiling

Microarray experiments were performed by Cogenics (Morrisville, NC, USA) using total RNA extracted from DCs. For each condition, three independent RNA extracts generated in one experiment were combined to ensure homogeneity of the samples. RNA samples were first assessed using a NanoDrop™ spectrophotometer and an Agilent bioanalyzer and then subjected to a 2-color standard Agilent amplification labeling method. cRNA labeled with Cy5 (control) and Cy3 (10^7^ or 10^4^ CFU) were synthesized using the Quick Amp kit (Agilent) with 500 ng of total RNA as a matrix. For each condition, 825 ng of Cy3- and Cy5-labeled cRNA were hybridized on an Agilent Whole Human Genome microarray 4×44K (Agilent). Data were extracted from washed and scanned microarrays using Feature Extraction software version 10.2 (Agilent). The results were analyzed using the BioRag database (BioResource for array genes, www.biorag.org). Biostatistical and bioinformatics evaluation of gene expression pattern was performed using the Resolver software (Rosetta Biosoftware). All the microarray data were deposited according to MIAME standards in the GeneExpression Omnibus (GEO) database at http://www.ncbi.nlm.gov/geo/, with the accession number GSE25699.

### qRT-PCR

For each sample, 1 µg of total RNA was reverse-transcribed for 50 min at 50°C using 200 U of SuperScript™ III Reverse Transcriptase and 50 pmol of oligo(dT)_20_ primer (Invitrogen). Resulting cDNA were diluted with two volumes (40 µl) of water and stored at −20°C. Quantitative polymerase chain reactions were performed using the LightCycler® FastStart DNA Master SYBR Green I kit as recommended by the manufacturer (Roche Applied Science, Meylan, France) with 1 µl of matrix cDNA. Primers are described in table S1. Reactions were performed in triplicate with three RNA samples extracted independently. Gene expression levels were normalized to those of the housekeeping genes *ACTB*, *RPLP0* and *TBP*, encoding respectively beta-actin, ribosomal protein P0 and TATA box binding protein.

### Maturation of the DCs in the presence of *L. rhamnosus* Lcr35 and analysis by flow cytometry

On day 6, the immature DCs from each well were harvested, pooled, centrifuged and reseeded at 1×10^5^ cells/mL. UV-killed bacteria were then added as 10 µL medium per well to a final concentration ranging from 10^3^ to 10^7^ CFU/mL, i.e. to a MOI ranging from 0.01 to 100. Lipopolysaccharide (LPS) from *Escherichia coli* (Sigma, Saint Quentin Fallavier, France) at a final concentration of 100 ng/mL was used as positive control. Immature dendritic cells without addition of LPS or bacteria were used as negative control. The cells were incubated at 37°C in 5% CO_2_ atmosphere. After 48 h of maturation, they were collected, centrifuged and resuspended in phosphate buffered saline (PBS) with 1% Bovine Serum Albumin (BSA, Sigma). Cell surfaces were stained with the appropriate fluorescence-labeled murine antibodies: APC-Cy7-conjugated anti-CD14 (specific molecule of the monocytes, co-receptor of the LPS), PE-conjugated anti-CD86 (co-stimulatory molecule, activation marker), FITC-conjugated anti-CD83 (specific marker of mature DCs whose biological functions are not yet clear), PE-conjugated anti-HLA-DR, PerCP-Cy5.5-conjugated anti-DC-SIGN (CD209, member of the CLR family, specific marker of immature DCs), Alexa488-conjugated anti-MR (Mannose Receptor or CD206, member of the CLR family, specific marker of immature DCs and macrophages) and streptavidin APC-conjugated anti-TLR4 (biotin antibody, a LPS receptor with activation functions). Antibodies were obtained from BD Biosciences (Le Pont de Claix, France) except anti-MR from Biolegend (Uithoorn, The Netherlands) and anti-HLA-DR from Beckman Coulter (Villepinte, France). Corresponding murine isotype-matched control antibodies (BD Biosciences and Biolegend) and non-labeled controls were performed. The cells were analysed using BD-LSRII with FACSDiva Software (BD Biosciences) from the Centre d'Imagerie Cellulaire Santé (CICS), Université d'Auvergne-Clermont1. Compensation fluorescence adjustments were performed. Gates were set on living DCs based on forward/side scatter properties. The analysis was based on a count of 3,000 DCs. The level of staining was expressed as the mean fluorescence intensity (MFI). The culture supernatant was collected and stored at −20°C until cytokine analysis. Each experiment was performed at least five times.

### Cytokine quantification

After 48 h of incubation, i.e. on day 8 after cells' harvest, the cytokines IL10 UltraSensitive, IL-12p40 and TNF-α were detected in culture supernatants from DC culture media using Biosource enzyme-linked immunosorbent assay (ELISA) kits (Nivelles, Belgium). At the same time, IL-1β and IL-23 were detected with Invitrogen ELISA Kits (France, Cergy-Pontoise) and IL-12p70 with eBioscience kit (San Diego, USA). All assays were performed according to the manufacturer's instructions.

### Statistical analysis

Student's matched pair t-test was used to compare means. P-values lower than 0.05 were considered statistically significant.

## Results

### Transcriptional profile of dendritic cells in response to probiotic *L. rhamnosus* Lcr35 by DNA microarray

All genes presenting a greater than 3-fold modification of their expression level were selected. Expression levels of 823 genes were increased and those of 859 genes were decreased after contact with 10^7^ CFU (MOI, 10) of *L. rhamnosus* Lcr35 bacteria. In contrast, the dendritic cells showed a weak transcriptional response after incubation with the probiotic at a MOI of 0.01 (10^4^ CFU), with only 58 genes upregulated and 138 downregulated. *In silico* analysis of the subcellular location of proteins encoded by the genes whose expression was modified in response to the lactobacilli revealed that, whatever the bacterial inoculum, most proteins belonged to the nuclear or the membrane compartments of the cells.

**Figure 1:**
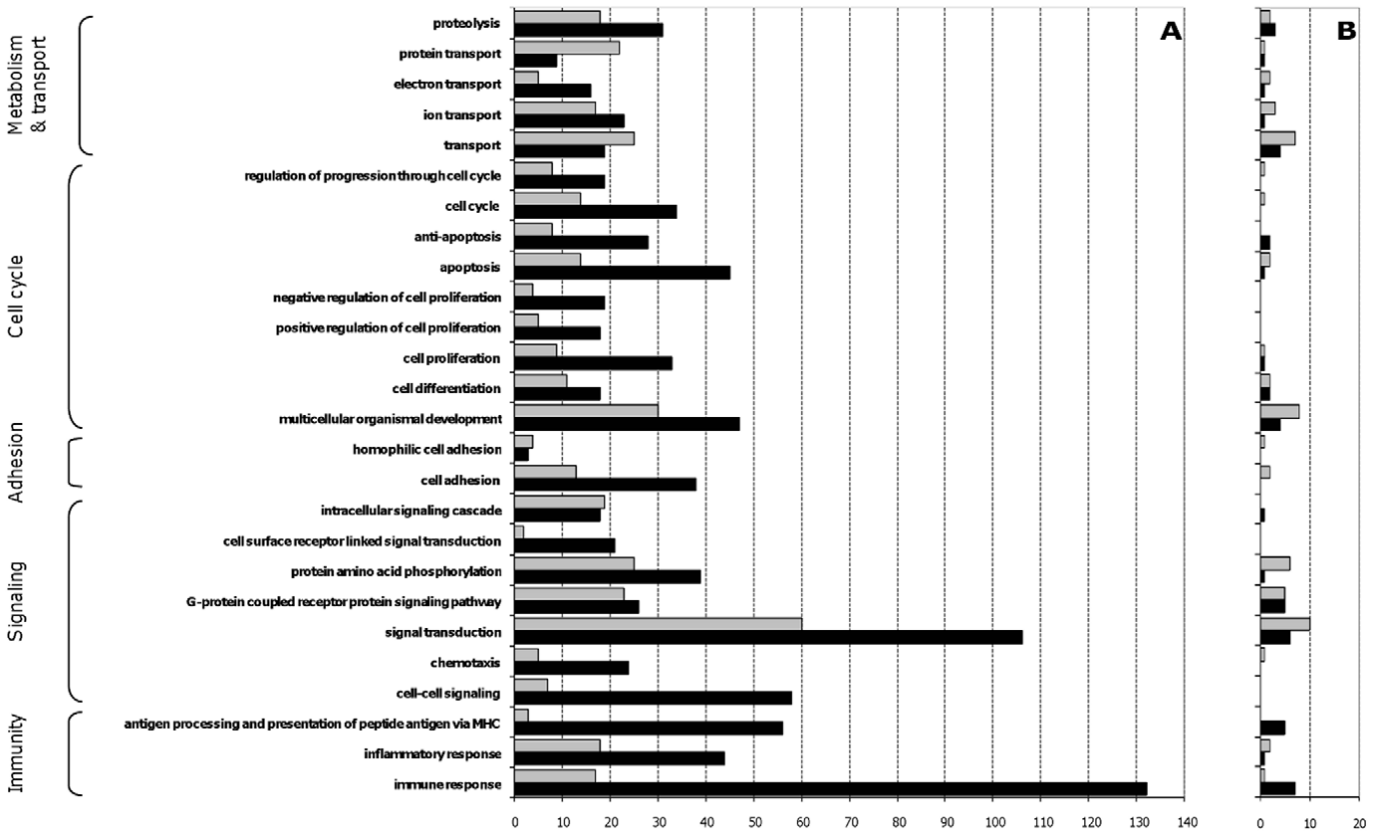
Most affected DC biological processes in response to a 3 h contact with *L. rhamnosus* Lcr35. The number of genes involved in each biological process (y axis) and whose expression level is increased (black) or decreased (grey) after contact with probiotic *L. rhamnosus* Lcr35 at a MOI of 10 (A) or 0.01 (B) is represented on the x axis.

Most of the genes whose expression was affected by contact with the probiotic at a MOI of 10 were involved in four main biological processes: 1/ immune and inflammatory responses, 2/ antigen processing and presentation via major histocompatibility complex class (MHC), 3/ intercellular signalling and 4/ signal transduction (figure 1A). Among the three first processes, almost all changes in expression displayed in response to *L. rhamnosus* Lcr35 were increases. Although most of the observed modifications concerning signal transduction were also gene upregulation, there were nonetheless a notable number of genes with lower transcriptional level. At lower MOI (0.01), the transcriptional response of dendritic cells was barely changed after contact with probiotic *L. rhamnosus* Lcr35 (figure 1B). Except for immune response, the modifications observed in the diverse processes were essentially repressions of gene expression and did not exceed 10-fold changes.

Comparison of our results with the Torri's model of molecular signature of inflammation indicated no significant effect in treated DCs at a MOI of 0.01. Conversely, at a MOI of 10, *L. rhamnosus* Lcr35 was considered as inducing a pro-inflammatory DC phenotype by 76% (41/54) of the genes, as inducing neither a pro-, nor an anti-inflammatory DC phenotype by 11% (6/54) and as inducing an anti-inflammatory DC phenotype by 13% (7/54) of the genes (table 1).

**Table 1:** Expression levels of DC genes allowing the comparaison with the molecular signature of inflammation by Torri *et al.* [9].

Gene	MOI = 0.01	MOI = 10	By Torri
***IL6***	1,090	20,661	up
***IL12B***	1,000	58,766	up
***PDZK1IP1***	1,672	1,246	up
***IL4I1***	1,033	1,742	up
***CCL22***	1,064	1,339	up
***CD83***	1,145	3,562	up
***GADD45B***	1,598	3,849	up
***IL1B***	1,077	177,495	up
***CD40***	0,966	1,907	up
***NFKBIZ***	1,341	5,051	up
***RAB20***	0,902	1,156	up
***CCR7***	1,112	4,953	up
***TRAF1***	0,956	14,687	up
***STAT5A***	1,014	2,638	up
***ISG15***	1,009	3,293	up
***NFKBIA***	0,998	1,750	up
***IRF8***	0,816	**0,874**	up
***ICAM1***	1,137	2,627	up
***NFKBIB***	1,224	1,904	up
***TNFRSF1B***	1,148	1,133	up
***SQSTM1***	0,998	1,025	up
***IFIT1***	0,887	1,748	up
***SKIL***	1,072	1,166	up
***GNAI3***	0,966	0,934	up
***USP18***	0,854	1,391	up
***NFKB1***	1,016	5,435	up
***CD86***	1,089	5,023	up
***CLIC4***	0,954	2,333	up
***TAP1***	0,865	1,478	up
***PSME2***	1,009	1,380	up
***ANXA4***	0,988	**0,569**	up
***DNAJB6***	0,978	1,361	up
***TRAFD1***	1,314	**0,580**	up
***SERPINB9***	0,817	8,334	up
***CDKN1A***	1,066	1,452	up
***DAXX***	1,227	1,931	up
***EIF4EBP2***	0,852	0,424	do
***ELOVL6***	1,385	0,732	do
***TM7SF3***	0,829	0,353	do
***TSPAN8***	1,000	1,000	do
***CCNB2***	0,900	0,791	do
***ZNRF2***	0,938	0,622	do
***LTA4H***	0,980	0,804	do
***IL18R1***	0,887	**8,427**	do
***TEP1***	0,886	0,751	do
***HDAC5***	1,221	0,554	do
***KIF20A***	1,106	**2,211**	do
***CDC20***	0,973	**1,976**	do
***DAGLB***	1,330	0,552	do
***SGPP1***	0,901	0,508	do
***MAN2B1***	1,464	1,090	do
***IL1RL1***	1,461	**5,450**	do
***HLA-DMA***	1,165	0,476	do
***TXNDC16***	1,007	0,499	do
**up : upregulation signs of inflammatory response according to Torri ** ***et al.***			
**do : downregulation signs of inflammatory response according to Torri ** ***et al.***			

The values discrepant with the expected response according to the model of Torri *et al.* are in bold.

In DC genes inducing the polarization of the T lymphocytes, i.e. mainly cytokine-encoding genes, no significant variation was observed with the lowest MOI (0.01) whatever the gene studied, whereas with a MOI of 10, great variations were observed (table 2). Thus, the expression of the genes directing to a Th1 (*IL12A*, *IL12B* and *TNF*) or a Th17 (*IL1B*, *IL6*, *IL23A*, *IL12B* and *TGFB1*) profile were strongly upregulated (fold-changes between 20 and 177), except *IL12A* and *TGFB1*. Expression of the DC genes directing to a Treg (*IL10*, *TGFB1*, *ALDH1A1* and *ALDH1A2*) or a Th2 (*IL4*, *IL2*, *IL7*, *TNFS4*) profile were either not modified (less than 3-fold change) or downregulated. Only IL-10 gene expression was increased, but sparsely (3.6-fold change) compared to pro-inflammatory cytokines-encoding genes.

**Table 2:** Expression levels of genes inducing the polarization of T lymphocytes after *L. rhamnosus* Lcr35-induced DC maturation, as determined by microarray.

DC-secreted molecule	Induced T cell	Gene	MOI = 0.01	MOI = 10
**TNF-α**	Th1	*TNF*	1.058	44.261
**IL-12p35** *	Th1	*IL12A* *	1.000*	1.000*
**IL-12p40**	Th1	*IL12B*	1.000	58.766
**IL-1β**	Th17	*IL1B*	1.077	177.495
**IL-6**	Th17	*IL6*	1.090	20.661
**IL-23p19**	Th17	*IL23A*	1.037	25.488
**IL-12p40**	Th17	*IL12B*	1.000	58.766
**TGFβ**	Th17	*TGFB1*	1.271	1.025
**IL-4**	Th2	*IL4*	0.664	1.175
**IL-2**	Th2	*IL2*	1.052	1.000
**IL-7**	Th2	*IL7*	0.749	1.381
**OX-40L**	Th2	*TNFS4*	1.553	2.724
**IL-10**	Treg	*IL10*	0.907	3.602
**TGFβ**	Treg	*TGFB1*	1.271	1.025
**retinoic acid**	Treg	*ALDH1A1*	0.974	0.548
**retinoic acid**	Treg	*ALDH1A2*	0.973	0.687

*unexpected data.

### qRT-PCR analysis of the probiotic effect on expression of target genes by dendritic cells

**Figure 2:**
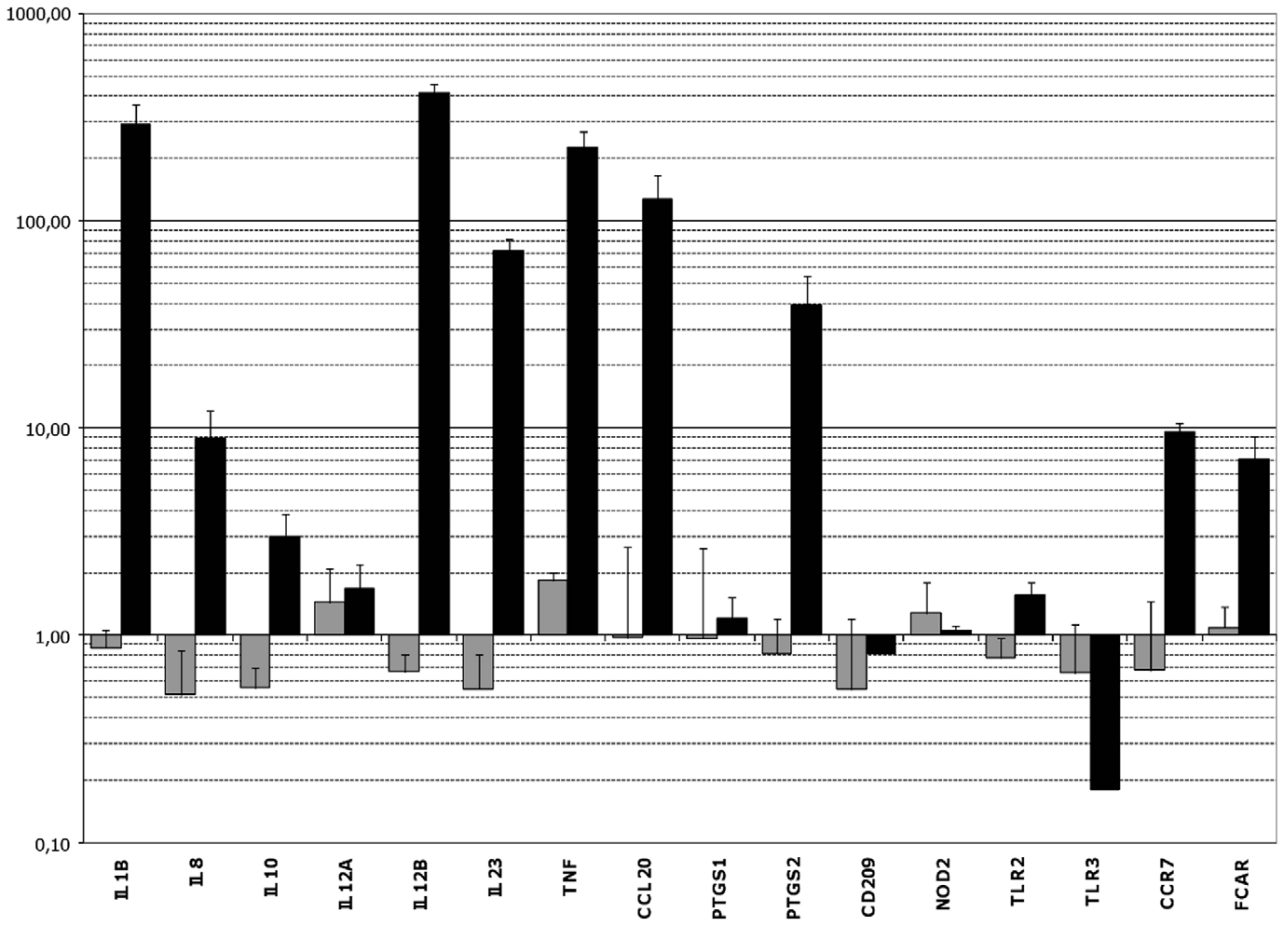
qRT-PCR analysis of target genes expression in DCs after contact with the probiotic Lcr35. Gene expression levels were determined by comparing samples exposed for 3 h to *L. rhamnosus* Lcr35 at a MOI of 0.01 (grey) or 10 (black) with a control sample exposed to PBS. The names of the target genes are indicated on the x axis. The results on the y axis correspond to geometric means (±standard error of the mean) of gene expression levels obtained from at least three independent samples.

Sixteen genes whose expression was especially affected after contact with strain *L. rhamnosus* Lcr35 in microarray assays and/or which seemed to be of particular interest for immune mucosal response were selected for further quantitative RT-PCR assays (table 3). None of the genes displayed a significant variation in their transcription level after exposure of the dendritic cells to probiotic *L. rhamnosus* Lcr35 at a MOI of 0.01 (figure 2). However, the slight modifications observed were mostly repressions of gene expression (*IL8*, *IL10*, *IL12B*, *IL23*). Conversely, with a larger bacterial inoculum (MOI, 10), transcription of genes *CCL20*, *IL1B*, *IL12B* and *TNF* was increased by about 100, 300, 400 and 200 fold respectively. These genes encode the CCL20 chemokine (especially chemotactic for memory lymphocytes and immature dendritic cells), the pro-inflammatory cytokines IL-1β and TNFα, and the IL-12p40 (or IL12β subunit, which is common to the IL-12 and IL-23 cytokines. The expression of *IL23* and *PTSG2* was also increased, although less strikingly (70 and 40-fold increase, respectively) and genes *CCR7*, *FCAR* and *IL8* were upregulated with a factor of about 9. The expression of the IL-10 encoding gene was multiplied by 3.01, but with standard error reaching 0.84 indicating therefore a lack of significance and reproducibility. The *TLR3* gene, encoding an intracellular receptor to double strand RNA, appeared to be repressed with its transcription levels being about 0.15 fold the one observed in basal conditions. Finally, no or little modification of expression was detected for the genes *IL12A*, *PTGS1*, *CD209*, *NOD2* and *TLR2*.

**Table 3:** Expression levels of genes of particular interest after *L. rhamnosus* Lcr35-induced DC maturation, as determined by microarray.

Genes	MOI = 0.01	MOI = 10
***IL1B***	1.08	**177.49**
***IL8***	1.15	**11.82**
***IL10***	0.91	**3.60**
***IL12A***	1.00	1.00
***IL12B***	1.00	**58.77**
***IL23A***	1.04	**25.49**
***TNF***	1.11	**30.96**
***CCL20***	0.81	**743.86**
***PTGS1***	0.99	0.45
***PTGS2***	0.97	**60.79**
***CD209***	1.03	0.67
***NOD2***	0.98	0.47
***TLR2***	1.06	**2.45**
***TLR3***	1.08	**0.08**
***CCR7***	1.11	**4.95**
***FCAR***	0.55	**6.55**

Values corresponding to an upregulation or a downregulation of at least 3 folds are in bold.

Overall, qRT-PCR analysis confirmed the variations in expression levels noticed in microarray hybridization assays resulting in an inflammatory signature in DCs treated with the highest dose of probiotic.

### Flow cytometric analysis of the probiotic effect on cell surface phenotype of dendritic cells

**Figure 3:**
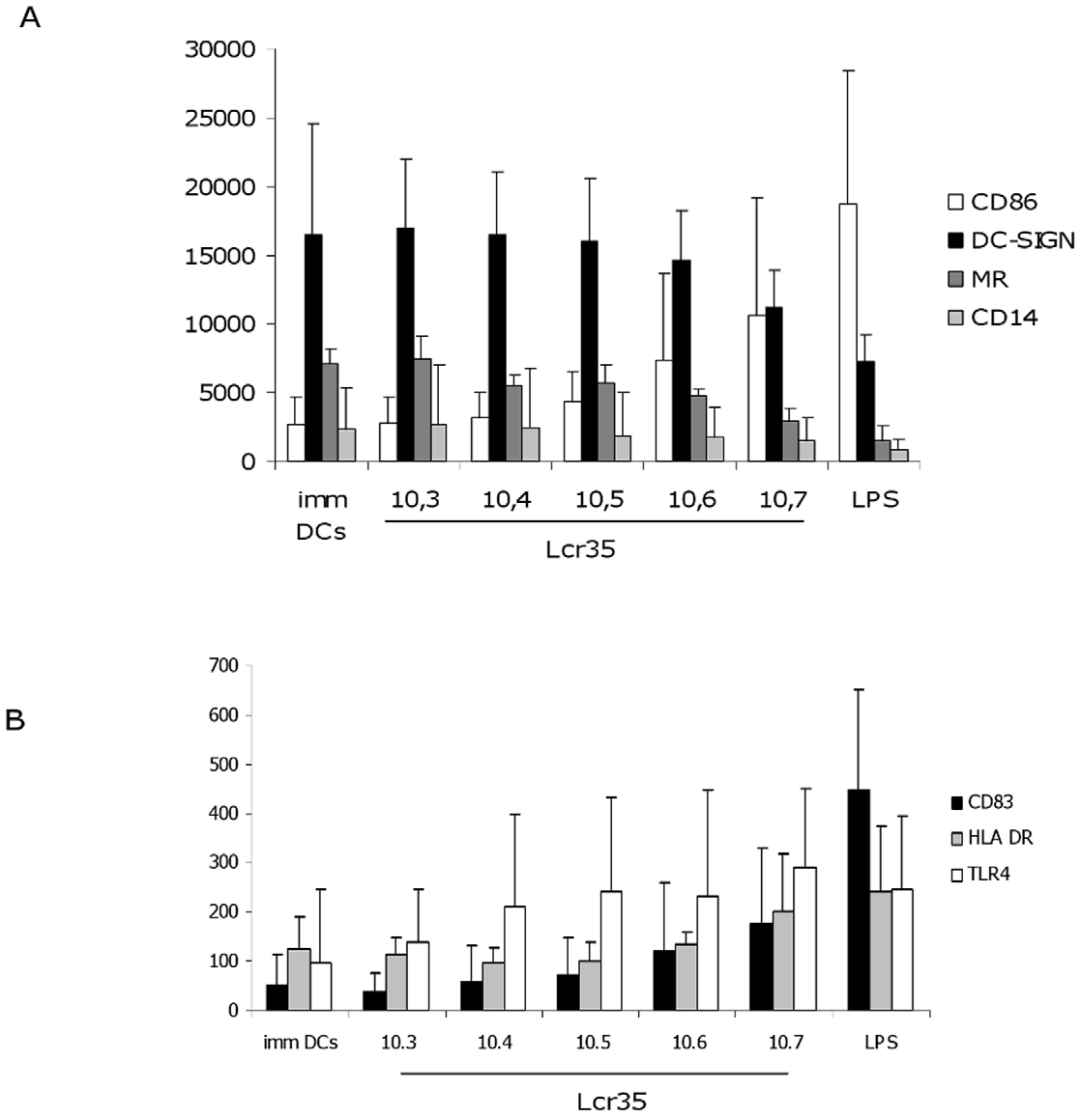
Maturation of human mo-DCs after exposure to a range of *L. rhamnosus* Lcr35 concentrations. The histograms show MFI values on gated DCs. Concentrations of *L. rhamnosus* Lcr35 ranged from 10^3^ to 10^7^ CFU/mL (MOI, 0.01, 0.1, 1, 10 and 100). (A) Differential expression on DCs of high density surface molecules CD86, DC-SIGN, MR and CD14. *L. rhamnosus* Lcr35 induced a dose-dependent higher expression of CD86 and a dose-dependent lower expression of DC-SIGN, MR and CD14 compared to immature DCs. (B) Differential expression on DCs of low density surface molecules : TLR4, HLA-DR, and CD83. *L. rhamnosus* Lcr35 induced a dose-dependent higher expression of CD83, HLA-DR and TLR4 compared to immature DCs. Data correspond to the mean and standard deviation of the MFI of 13 (A), and 5 (B) representative experiments. Isotypic control values were always subtracted from the MFI of each marker. imm DCs, immature DCs; Lcr35, DCs matured with *L. rhamnosus* Lcr35 at different concentrations; LPS, DCs matured with extracts of *E. coli* LPS.

DC maturation was assessed by changes in an extensive membrane phenotype. As expected, immature DCs were characterized by high levels of DC-SIGN and MR expression, together with low expression of CD86 and CD14 (CD14 was compared with the initial expression on monocytes before differentiation of DCs, data not shown) (figure 3A) and low expression of CD83, HLA-DR and TLR4 (figure 3B). Upon maturation, *L. rhamnosus* Lcr35 induced a dose-dependent higher and lower expression of CD86 and of DC-SIGN, MR and CD14, respectively (figure 3A). Low-dose probiotics maintained immature DC cell surface phenotype: at MOI of 0.01 (10^3^ CFU/mL), CD86, DC-SIGN, MR and CD14 surface expression was not modified. In contrast, at MOI of 0.1 (10^4^ CFU/mL), CD86, DC-SIGN, MR and CD14 levels were slightly modified, reflecting very early activation, which rose at higher bacterial concentrations to a MOI of 100 (10^7^ CFU/mL). At this concentration, CD86 expression was clearly upregulated whereas those of DC-SIGN and MR were manifestly downregulated (figure 3A). For markers with a lower cell surface density, such as CD83, HLA-DR and TLR4, *L. rhamnosus* Lcr35 also induced a dose-dependent higher surface expression as detected by flow cytometry (figure 3B). These results closely corroborate those obtained by studying DC gene expression with regard to high surface density markers (4/4), but not for low dose density markers (1/3) (table 4). As expected, in the control experiment, LPS-induced DCs expressed a phenotype, called fully mature DCs, with a very high level of CD86, decreased levels of DC-SIGN, MR and CD14 (figure 3A) associated with increases in the synthesis of markers with low surface density such as CD83, HLA-DR and TLR4 (figure 3B). The *L. rhamnosus* Lcr35-induced phenotype was thus intermediate between that of immature DCs and that of fully mature LPS-induced DCs, a so-called semi-mature DCs phenotype.

**Table 4:** Expression levels of DC genes at a MOI of 10, as determined by microarray, compared to MFI variations of membrane markers detected by flow cytometry.

Gene	Microarray (Fold changes)	Cytometry data (MFI)	
***CD86***	5.023	hi	up
***CD209-DC-SIGN***	0.536	hi	do
***MRC1L1-CD206***	0.361	hi	do
***CD14***	0.325	hi	do
***CD83***	3.562	lo	up
***HLA-DRA***	**0.456**	lo	**up**
***TLR4***	**0.423**	lo	**up**
**hi : marker of high membrane density**			
**lo : marker of low membrane density**			
**up : upregulation of the cytometric marker**			
**do : downregulation of the cytometric marker**			

The discrepant values between the two techniques are in bold.

### Cytokine production induced by *L. rhamnosus* Lcr35-matured DCs

**Figure 4:**
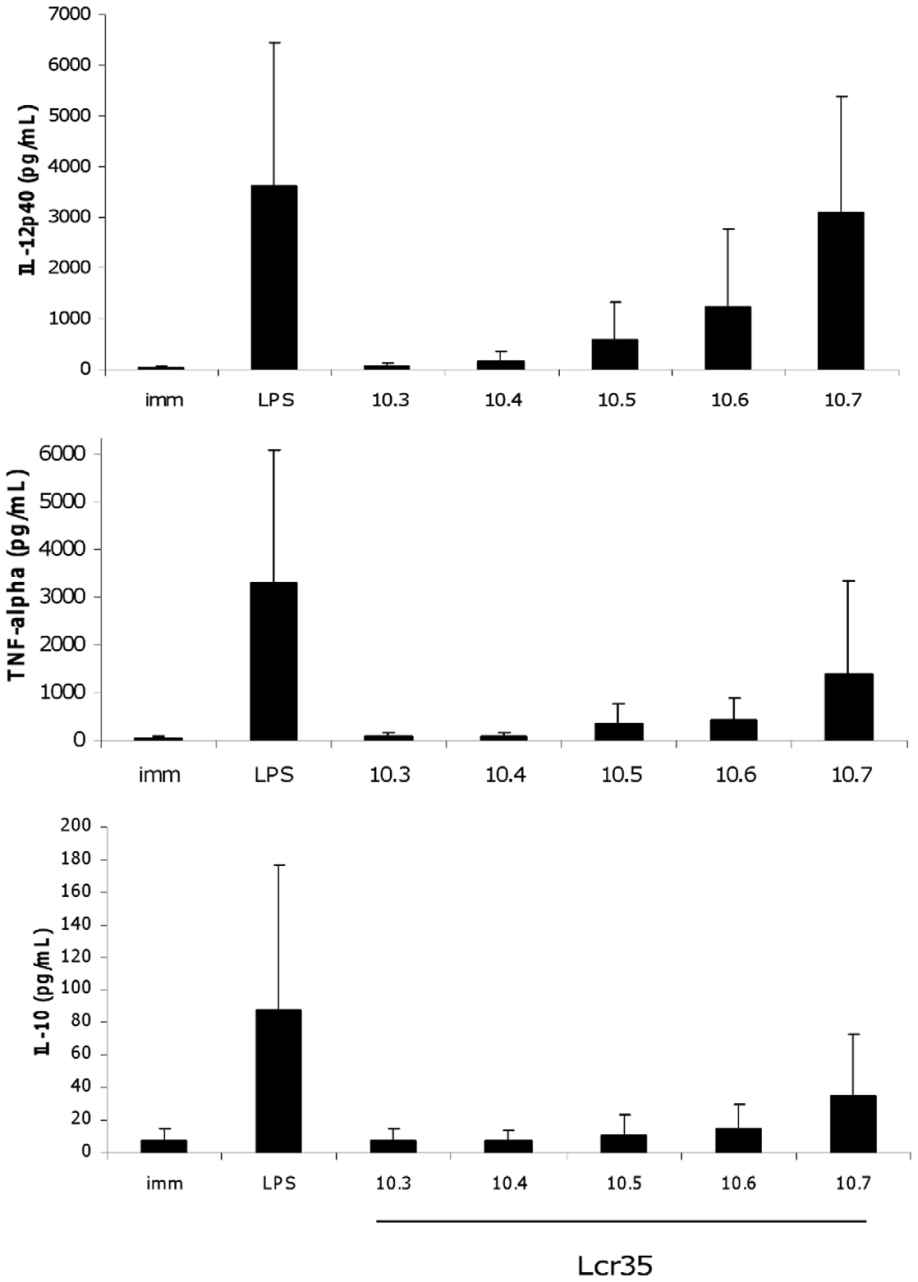
Cytokine production of human mo-DCs after 48 h exposure to a range of *L. rhamnosus* Lcr35 concentrations. Concentrations of *L. rhamnosus* Lcr35 ranged from 10^3^ to 10^7^ CFU/mL (MOI, 0.01 to 100). *L. rhamnosus* Lcr35 induced a dose-dependent production of IL-12p40, TNF-alpha and IL-10. The production of IL-12 and TNF-alpha pro-inflammatory cytokines was much higher than that of IL-10. Data correspond to the mean and standard deviation of five independent experiments.

Six cytokines whose encoding gene expression was especially affected by contact with strain *L. rhamnosus* Lcr35 in quantitative RT-PCR assays and/or which seemed to be of particular interest for the understanding of the DC response were selected for ELISA measurements. Compared to untreated immature DCs, a strong dose-dependent increase in the production of IL-12p40 and TNF-α was induced by *L. rhamnosus* Lcr35 and, to a lesser extent, in the production of IL-10 (figure 4). The fold changes determined by comparing the level of cytokines produced by 10^7^ CFU/mL *L. rhamnosus* Lcr35-treated DCs (i.e MOI, 100) with that of immature DCs were respectively 73 for IL-12p40, 38 for TNF-α and, merely, 4 for IL-10 production. These great differences are also in favour of a strongly pro-inflammatory profile induced by the probiotic at high concentrations.

**Figure 5:**
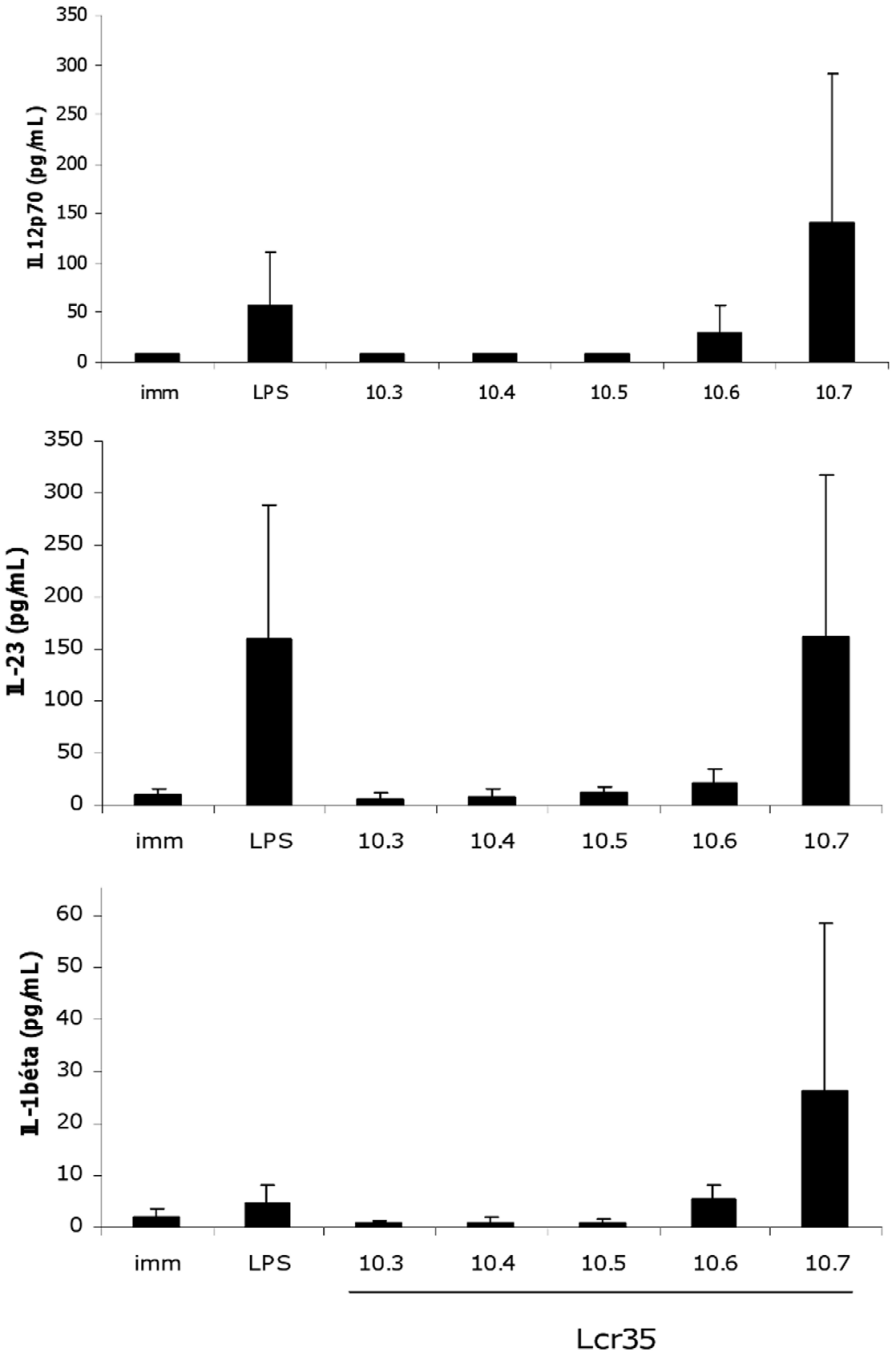
Cytokine production of human mo-DCs after 48 h exposure to a range of *L. rhamnosus* Lcr35 concentrations. Concentrations of *L. rhamnosus* Lcr35 ranged from 10^3^ to 10^7^ CFU/mL (MOI, 0.01 to 100). *L. rhamnosus* Lcr35 induced a dose-dependent production of IL-12p70 (pro-Th1), IL-23 (pro-Th17) and IL-1β (pro-Th17). Data correspond to the mean and standard deviation of six independent experiments.

Since IL-12p40 is the common subunit shared by IL-12p70 (or IL-12) and IL-23, we next examined their specific levels (figure 5). IL-12p70 was not or barely detectable in immature DCs and in *L. rhamnosus* Lcr35-treated DCs at MOI ranging from 0.01 to 1 (from 10^3^ till 10^5^ CFU/mL). At MOI of 10 (10^6^ CFU/mL), this cytokine became detectable and, at MOI of 100 (10^7^ CFU/mL), its concentration exceeded the LPS-induced level (MOI,100/LPS-induction, fold change = 3). IL-23 was also not or barely detectable in *L. rhamnosus* Lcr35-treated DCs with MOI ranging from 0.01 to 1 (figure 5). At higher *L. rhamnosus* MOI, the concentrations of IL-23 increased, but stayed at the level of LPS-treated DCs (MOI,100/LPS-induction, fold change = 1). Finally, we performed IL-1β measurements. The protein was once again not detectable at low concentrations, but was produced with the highest MOIs (MOI, 10 or 100), in agreement with the data from molecular analysis (microarray and RT-PCR). The combined increases of TNF-α, IL-12p40, IL-12p70, IL-1β and IL-23 indicated that *L. rhamnosus* Lcr35 induced a pro-Th1/Th17 DC phenotype.

## Discussion

Probiotics have been shown to exert health benefits, mostly in the prevention and treatment of enteric infectious and inflammatory diseases [18]–[21]. However, understanding how these bacteria contribute to human health remains a major challenge. One main difficulty is to assess their interactions with components of the intestinal immune system, especially the DCs, a key player in mucosal immunity. Specific probiotic strains, in particular *Bifidobacterium* and *Lactobacillus* strains, have been shown to interact with DCs and to induce strain-specific effects. Indeed, great variations in the ability of different strains of *Lactobacillus* to induce DC production of key cytokines such as IL-12 and IL-10 were observed, resulting in either pro-inflammatory or, conversely, anti-inflammatory patterns [22]. However, there are great differences between studies in bacteria/DCs ratio. The eventuality that these variations of MOI affected the DCs maturation process, and therefore their response to the probiotic strains, cannot be excluded [14], [23]–[27].

*Lactobacillus rhamnosus* Lcr35 is a well characterized probiotic strain which has been used in clinical practice for more than 50 years. The bacteriological effects of the strain have been documented in the past decade [15], [28], [29] but little is known about its interactions with the immune system. To provide insight into the immunological properties of the specific probiotic *L. rhamnosus* Lcr35 strain, we studied its interactions with immature DCs, using a large range of bacterial concentrations and both genomic and phenotypic approaches.

Gene array analysis is a new approach to evaluate the probiotic effects on immune cells [30], which provided an overall view of the changes in gene expression patterns of probiotic-matured DCs resulting from contact with different bacterial concentrations. Our results showed great dose-dependent variations. At a MOI of 10, the overexpression of 823 genes (with a 3-foldchange threshold) most of which are involved in the immune response was observed together with a decrease in the expression of 859 other genes, whereas at a lower MOI (0.01), the expression of only 139 genes was modified. In addition, the analysis of the gene expression profile according to the model of Torri *et al.* identified an inflammatory signature in DCs treated with the highest dose of probiotic, whereas at lower MOI, *L. rhamnosus* Lcr35 induced neither anti-inflammatory nor pro-inflammatory effects. The very different profiles obtained according to the bacterial inocula in this work should lead us to be more cautious in the interpretation of *in vitro* data when a single dose of probiotics is used. This also raises the question of what happens *in vivo*. Most commercial preparations of probiotics are taken orally and little is known about the remaining concentration of probiotics in contact with the mucosal DCs when they reach the small intestine or the colon. Depending on the doses ingested and their frequency, the effects induced by the probiotics could be very different and suitable for the treatment of different diseases, needing to be fought by pro- or anti-inflammatory responses.

Analysis of the genes involved in the orientation of the T cell response using microarray data revealed that *L. rhamnosus* Lcr35 was able to induce a strong pro-Th17 DC response. Of the five pro-Th17 genes tested, only the TGFβ gene was not upregulated, but, unlike in murine models, the role of TGFβ in Th17 induction in humans is still a contentious issue [31]. Similarly, qRT-PCR analysis of the genes indicated that *L. rhamnosus* Lcr35 at high concentrations was strongly pro-inflammatory, with a major pro-Th17 DC type of response. For the pro-Th1 response, contradictory results were obtained in microarray analysis regarding the two subunits of IL-12, with an increase in IL-12p40 gene expression (shared by IL-12 and IL-23) but no variation in IL-12p35 (specific of IL-12) messenger level. The upregulation of IL-12p40 expression could be corroborated by the strong increase in the expression of the TNF-α encoding gene, but the absence of variation, whatever the MOI, for the IL12p35-encoding gene was rather unexpected. The qRT-PCR results regarding the pro-Th1 genes were in agreement with these findings, with a strong increase in IL-12p40 and TNF-α mRNAs, but only a low increase in the case in IL-12p35.

Transcription analysis data was further completed by the determination of the DCs phenotype, including analysis of cytokine production. The upregulated production of TNF-α, IL-1β, IL-23, IL-12p40 and IL-12p70 cytokines as measured by ELISA showed that this probiotic effectively enhanced pro-inflammatory and pro-Th17 immune responses, but also pro-Th1 response. Lcr35 therefore behaves like many species of lactobacilli, *L. casei*, *L. gasseri*, *L. johnsonii*, *L. reuteri*, and *L. rhamnosus* GG, previously described as pro-Th1 inducers [23], [32], [33]. To our knowledge, this is the first time a probiotic *Lactobacillus* has been reported to have a pro-Th17 effect on DC maturation, but this was not tested in previous studies. Obtaining a combined response both pro-Th1 and pro-Th17 is not surprising, because Th1 and Th17 responses are two types of pro-inflammatory response which may be combined and acte together in the response against many pathogens [34].

Niess *et al.* recently reported that in mice, enteric flora induced the expansion of a specific type of lamina propria DCs, the CX3CR1^+^ DCs, which led to a mucosal inflammatory response and preferentially induced Th1/Th17 T cell differentiation [35]. These DCs are CD14^+^ and derive from monocytes under the control of GM-CSF [36], [37]. It is therefore tempting to postulate that the *L. rhamnosus* Lcr35 strain-induced DCs are related to these murine DCs. Indeed, in our experiments, human *L. rhamnosus* Lcr35-treated DCs were generated *in vitro* from monocytes by culturing PBMCs with GM-CSF (and IL-4) [38]–[39], and were phenotypically CD14^+^. In addition, as CX3CR1^+^ DCs, they supported Th1/Th17 T cell differentiation.

In the murine model, induction of Th1/Th17 cells by CX3CR1^+^ DCs initiates the host defence against intestinal pathogens, such as *Salmonella* [40]. *L. rhamnosus* Lcr35, by acting on DCs and facilitating Th1/Th17 T cell differentiation, could therefore indirectly participate in the defense against pathogens. Elsewhere, the main clinical indications of *L. rhamnosus* Lcr35 probiotic are related to its anti-infectious properties, in particular in infectious diarrhea and in microbial vaginosis [17], [41], [42]. Our results are based on *in vitro* experiments and need to be assessed in *in vivo* studies. In addition, our flow cytometry data indicated that *L. rhamnosus* Lcr35 induced semi-maturation of DCs with an upregulation of the membrane expression of HLA-DR, CD86 and CD83, combined with upregulation of CCR7 observed in qRT-PCRs. Taken together, these results demonstrate that, compared to immature DCs, probiotic-treated DCs have a higher potential of antigen presentation, costimulation and migration, and therefore should have a greater ability to induce an immune response of an effector type, as required when encoutering pathogens.

The Lcr35 probiotic might also be useful in the treatment of allergic disorders. A recent work showed that oral treatment with Lcr35 prior to sensitization can attenuate airway inflammation and hyperreactivity in a murine model of allergic airway inflammation, suggesting that Lcr35 has the potential for preventing asthma [43]. In recent years, many studies on the use of probiotics in the prevention or treatment of allergy have focused on their role, by acting on monocytes, PBMCs or DCs, in the production of IL-10 and in the generation of regulatory cells [13], [44]–[47]. Smits *et al.* demonstrated that both *L. reuteri* and *L. casei*, but not *L. plantarum*, bind the DC-SIGN on membrane DCs and that this ligation can actively prime DCs which produced IL-10 to induce Treg cells. As *L. rhamnosus* Lcr35 induced only a very low increase in IL-10 and only at high concentrations, and as it did not modulate the gene expression of TGFβ and of the enzymes allowing retinoic acid synthesis, it is very unlikely that it induced Tregs. Thus, the anti-allergic mechanism of action of *L. rhamnosus* Lcr35 probably does not involve the Tregs regulatory pathway. Since *L. rhamnosus* Lcr35 modulates the Th1/Th2 balance towards the Th1(/Th17) response in DCs, it could counter-regulate the impaired cytokine profile observed in IgE-dependent allergies, characterized by an imbalance of the Th1/Th2 response towards the Th2 profile. Future clinical studies are necessary to investigate this possibility.

Combined, our findings show that the probiotic *L. rhamnosus* Lcr35 induces a dose-dependent immunomodulation of human DCs leading, at high bacterial doses, to the semi-maturation of the cells and a strong synthesis of pro-Th1/Th17 cytokines. These results open up broader prospects regarding the clinical indications in which this probiotic could be used, to strengthen the immune defences in the case of infections or in the immunomodulation of IgE-dependent allergic diseases.

## Supporting Information

Figure S1 **Cytometric analysis of the DC-SIGN expression on the DCs (MFI), gated on the FSC/SSC dot plot.** The purity of the DCs, evaluated as the percentage of cells expressing the DC-SIGN, was always above 90%.
(PPT)

Table S1 **Primers used in qRT-PCRs.**
(DOC)
