# Supplementary figures and images for: Dose-Dependent Immunomodulation of Human Dendritic Cells by the Probiotic Lactobacillus rhamnosus Lcr35
Source: PLoS One. 2011 Apr 18;6(4):e18735. doi: 10.1371/journal.pone.0018735 (PMC3078917; doi:10.1371/journal.pone.0018735)

## Slide 1
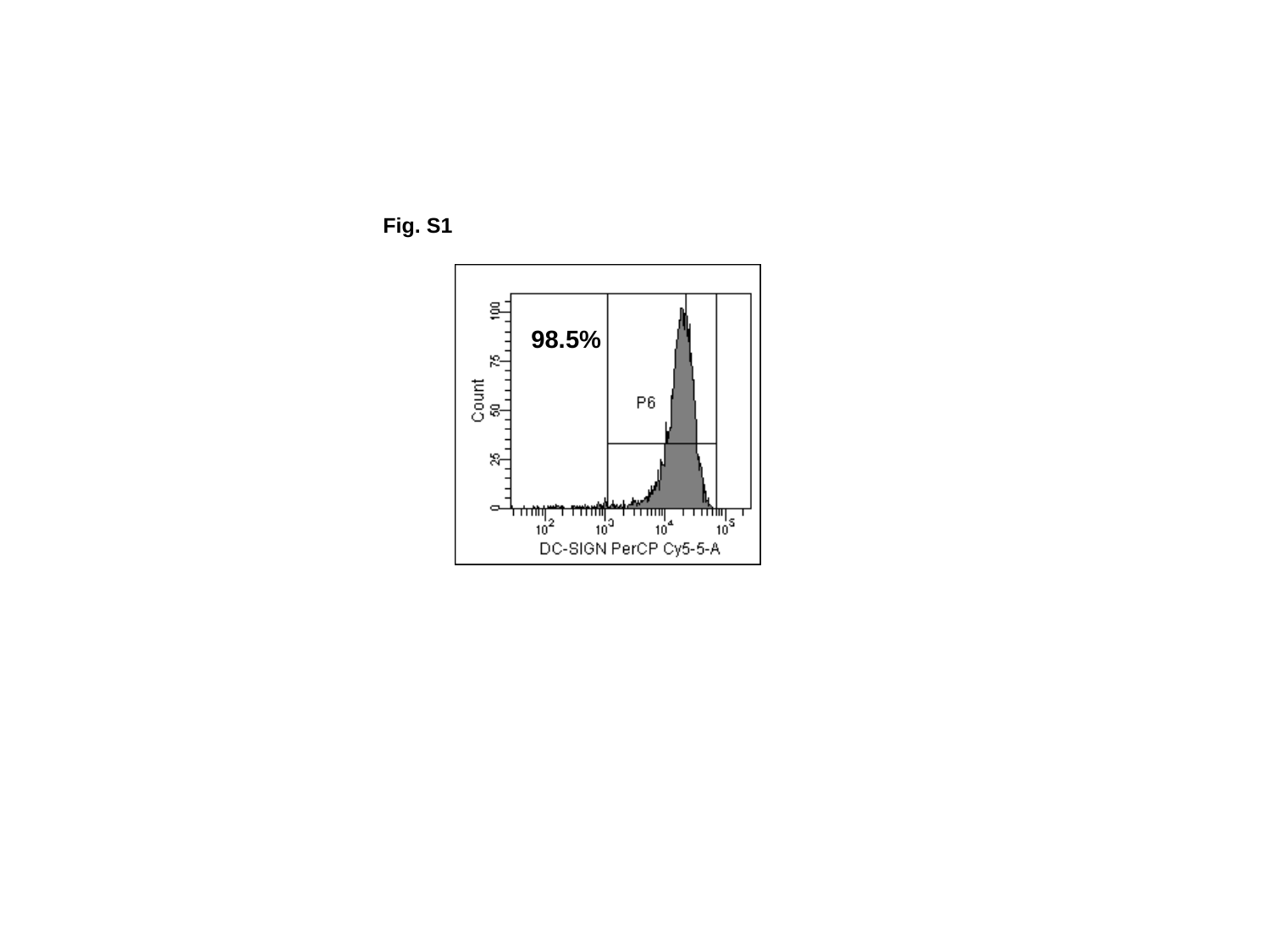

Fig. S1
98.5%

Supplement: Figure S1 — Cytometric analysis of the DC-SIGN expression on the DCs (MFI), gated on the FSC/SSC dot plot. The purity of the DCs, evaluated as the percentage of cells expressing the DC-SIGN, was always above 90%. (PPT) [file pone.0018735.s001.ppt]
