# Supplementary material for: Dose-Dependent Immunomodulation of Human Dendritic Cells by the Probiotic Lactobacillus rhamnosus Lcr35
Source: PLoS One. 2011 Apr 18;6(4):e18735. doi: 10.1371/journal.pone.0018735 (PMC3078917; doi:10.1371/journal.pone.0018735)
Supplement: Table S1 — Primers used in qRT-PCRs. (DOC) [file pone.0018735.s002.doc]

**Table S1: Primers used in qRT-PCRs**

**Target Sequence (5’🡪3’) amplicon**

**gene forward primer reverse primer size (bp)**

***ACTB*** ATGTGGCCGAGGACTTTGATT AGTGGGGTGGCTTTTAGGATG 107

***RPLP0*** GCGGTTTCTGATTGGCTACTT AAGACGATGTCACTTCCACGA 98

***TBP*** CTCCCCCACCCCCTTCTTT CCACACCCTGCAACTCAACA 103

***CCL20*** GTGCTGCTACTCCACCTCTG CGTGTGAAGCCCACAATAAA 110

***CCR7*** ACAGCCTTCCTGTGTGGTTT TCTCCGATGTAATCGTCCGT 142

***CD209*** AGCAGAACTTCACCCCCTTT GGAGGCATGACAAGAAGGAC 139

***FCAR*** CTTCCTGCGGAGCTTATTGT ACAGGAGGGTGGTCTGTTTG 119

***IL1B*** AGCTGATGGCCCTAAACAGA AAGCCCTTGCTGTAGTGGTG 108

***IL8*** CCGGAAGGAACCATCTCACT AGCACTCCTTGGCAAAACTG 117

***IL10*** GTGGAGCAGGTGAAGAATGC GCCACCCTGATGTCTCAGTT 137

***IL12A*** CCCATGCCTTCACCACTCCC GGCCTCCACTGTGCTGGTTT 158

***IL12B*** GGTATCACCTGGACCTTGGA GCTTAGAACCTCGCCTCCTT 126

***IL23*** AGAAGCTCTGCACACTGGC CCACACTGGATATGGGGAAC 109

***NOD2*** CAACAACAAATTGACTGACGGCT CAGGAACTGCAAGGAGGTGTT 160

***PTGS1*** CCGGAGTCTCTTGCTCTGGT CTGGTGCTGGCATGGATAG 124

***PTGS2*** GGTGGAGAAGTGGGTTTTCA CGGGAAGAACTTGCATTGAT 146

***TLR2*** GGCAAAATCATTTGGCATCATTGG CACTGACAAGTTTCAGGCATA 118

***TLR3*** AAAGGAAAGGCTAGCAGTCATC ATGCACACAGCATCCCAAAG 99

***TNF*** TCAGCCTCTTCTCCTTCCTG GAGAAGATGATCTGACTGCCT 147
